# vi-HMM: a novel HMM-based method for sequence variant identification in short-read data

**DOI:** 10.1186/s40246-019-0194-6

**Published:** 2019-02-13

**Authors:** Man Tang, Mohammad Shabbir Hasan, Hongxiao Zhu, Liqing Zhang, Xiaowei Wu

**Affiliations:** 1 Department of Statistics, Virginia Tech, 250 Drillfield Drive, Blacksburg, 24061 VA USA; 2 Department of Computer Science, Virginia Tech, 225 Stanger Street, Blacksburg, 24060 VA USA

**Keywords:** HMM, Variant calling, SNP, INDEL, Viterbi algorithm

## Abstract

**Background:**

Accurate and reliable identification of sequence variants, including single nucleotide polymorphisms (SNPs) and insertion-deletion polymorphisms (INDELs), plays a fundamental role in next-generation sequencing (NGS) applications. Existing methods for calling these variants often make simplified assumptions of positional independence and fail to leverage the dependence between genotypes at nearby loci that is caused by linkage disequilibrium (LD).

**Results and conclusion:**

We propose vi-HMM, a hidden Markov model (HMM)-based method for calling SNPs and INDELs in mapped short-read data. This method allows transitions between hidden states (defined as “SNP,” “Ins,” “Del,” and “Match”) of adjacent genomic bases and determines an optimal hidden state path by using the Viterbi algorithm. The inferred hidden state path provides a direct solution to the identification of SNPs and INDELs. Simulation studies show that, under various sequencing depths, vi-HMM outperforms commonly used variant calling methods in terms of sensitivity and *F*_1_ score. When applied to the real data, vi-HMM demonstrates higher accuracy in calling SNPs and INDELs.

**Electronic supplementary material:**

The online version of this article (10.1186/s40246-019-0194-6) contains supplementary material, which is available to authorized users.

## Introduction

Rapid evolution of next-generation sequencing (NGS) technologies in recent years enables various genetic applications in a fast, efficient, and cost-effective way [1, 2]. One fundamental procedure in NGS data analysis is variant calling, i.e., to identify the existence of genetic variants from short-read data. Accurate and reliable identification of single nucleotide polymorphisms (SNPs) and insertion-deletion polymorphisms (INDELs) plays an important role in all NGS applications as these common sequence variants are highly abundant in the human genome and have been found to likely influence human traits and disease [3–5].

The process of variant calling starts with aligning a set of short reads to the reference genome. After reads are correctly mapped, statistical models or heuristics may be used to predict the likelihood of variation at each locus based on available information such as quality scores and allele counts of aligned reads at the locus [6]. Most statistical models used for variant calling are built on the Bayes’ theorem, with an ultimate goal to predict genotypes from aligned reads by using the maximum a posteriori (MAP) estimate. Following this Bayesian approach, a number of variant calling tools have been developed, including SAMtools [7], GATK [8], FreeBayes [9], and Platypus [10]. Heuristic-based tools, such as VarScan [11], call variants based on a variety of heuristic factors, e.g., minimum allele counts, read quality cut-offs, and bounds on read depth. Though heuristic methods could be robust to outlier data that do not follow probabilistic model assumptions, the selection of cutoffs and bounds is highly empirical which largely restricts their practical usage. Other alternatives such as machine learning tools [12] are also applicable for variant calling, but they appear to be relatively unpopular in practice. Due to divergence of the model assumptions, these variant calling tools perform quite differently on NGS data [13, 14]. It should be noted that, although Bayesian statistical models are highly prevalent in variant calling, existing tools developed using this approach often make simplified assumptions of positional independence and fail to leverage the dependence between genotypes at nearby loci that is caused by linkage disequilibrium (LD). A statistical model that appropriately incorporates such dependence information has the potential to improve the accuracy of variant detection, especially in regions of high LD in the human genome.

Hidden Markov models (HMMs) can effectively model dependence between adjacent symbols or regions, thus have been extensively used in various disciplines [15]. Since its first application in computational biology in the late 1980s [16], HMMs become popular in biological sequence analysis [17]. Generally speaking, the occurrences of genetic variants (SNPs and INDELs) on the genome are not independent events because of the existence of LD between SNPs or between INDELs and SNPs [4, 18]. For this reason, one may use Markov models to better characterize the dependence between genotypes at nearby loci in order to improve the analysis of NGS data. Several HMM-based programs have been developed for read mapping and variant calling in sequencing data, including Dindel [19], PyroHMMsnp [20], and PyroHMMvar [21]. All these programs call SNPs and/or INDELs by estimating top candidate (most likely) haplotypes/genotypes using the Bayesian approach. In particular, Dindel [19] constructs a two-layer HMM by treating both the insertion status and its position index as hidden variables, and PyroHMMsnp [20] and PyroHMMvar [21] use HMMs to formulate homopolymer errors and employ a weighted alignment graph to reconstruct the consensus sequences. Though these programs show remarkable flexibility in detecting genetic variants, they are usually designed for specific applications: Dindel is for INDEL calling only, and PyroHMMsnp and PyroHMMvar emphasize the modeling of homopolymer. Moreover, the Bayesian paradigm of these programs may slow down the variant calling process when dealing with massive datasets.

In this paper, we propose vi-HMM, a novel HMM-based method for identifying small-scale sequence variants in short-read data. This method allows transitions between hidden states (hereafter defined as “SNP,” “Ins,” “Del,” and “Match”) of adjacent genomic loci and determines an optimal hidden state sequence by using the Viterbi algorithm. The inferred hidden state sequence provides a direct solution to the identification of SNPs and INDELs. Through simulations, we show that vi-HMM represents an improvement over five other variant callers—GATK HaplotypeCaller, FreeBayes, Platypus, SAMtools, and VarScan in terms of sensitivity, precision, and *F*_1_ score. When applied to a real short-read dataset (NA12878) generated by the Genome in a Bottle (GIAB) project [22], vi-HMM demonstrates its major advantage in identifying INDEL variants as compared to four other variant callers—FreeBayes, Platypus, SAMtools, and VarScan, while still maintaining good performance in SNP calling at different read coverage depths.

## Methods

Along the genome, the states of genomic bases, i.e., whether or not and which type of sequence variants exist on the bases, often exhibit dependence. Incorporating such dependence information helps improve the accuracy of variant calling but poses challenges in calculating the joint likelihood of the entire sequence. In this study, we assume Markov property for the dependence and accordingly propose a new method for *variant identification on the basis of HMM*, acronymized by vi-HMM.

The vi-HMM method performs variant calling for SNPs and INDELs after short reads are mapped to a reference genome (an example of the mapped reads is shown by the IGV visualization tool in Additional file 1). Its input includes a reference genome sequence and a file with mapped reads (a SAM/BAM file). The core of this method lies in the construction of an HMM that models state transitions among the bases on the genome as well as emissions from the hidden states to the observed pileup read data. From the HMM, we can uncover the optimal hidden state sequence (i.e., the Viterbi path), which is then used to call variants or infer the underlying genotypes. The workflow of the vi-HMM algorithm is shown in Fig. 1, including three major steps:

1. Define the states (Match, SNP, Insertion, and Deletion) and identify the transition probabilities among the states to build the transition matrix.

**Fig. 1:**
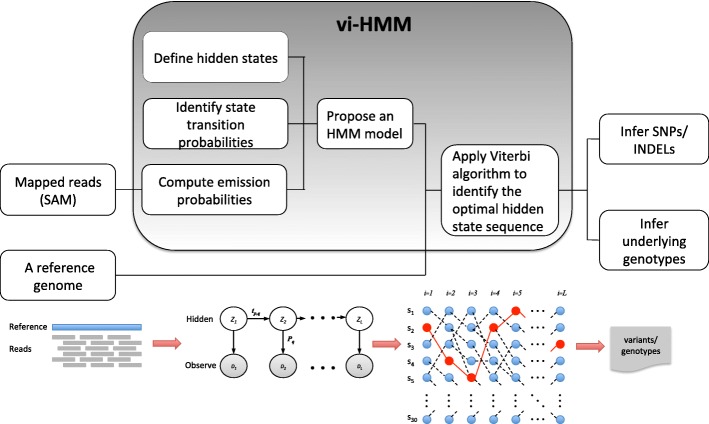
Workflow of vi-HMM
2. Compute the likelihood (emission probability) of observing the pileup of reads under different states.
3. Given a reference genome sequence, find the optimal hidden state sequence by using the Viterbi algorithm and based on which infer variants/genotypes.

Details of these steps are explained in the following subsections.

### The states and transition matrix

We assume that all reads have been mapped to the reference genome by a standard mapping tool, such as Bowtie2 [23] or BWA-MEM [24], resulting in a SAM file. Using the CIGAR strings from the SAM file, detailed alignment information for each base can be obtained, which indicates the relation between the reference genome and the genotype sequence that underlies the mapped reads. We consider a genomic region with length *L*, that is, a total of *L* adjacent bases including the ones in the reference but not in the genotype and vice versa. We define an alphabetic set *Ω*={*A*,*C*,*G*,*T*,−} to include the symbolic elements in this genomic region with *A*,*C*,*G*,*T* denoting the nucleotides and “ −” denoting a missing nucleotide caused by deletions or insertions. Let *R*_*i*_ and *G*_*i*_, 1≤*i*≤*L* denote the symbol on base *i* for the reference sequence and for the genotype sequence, respectively. Then, *R*_*i*_∈*Ω*, and *G*_*i*_ can take 15 possible diploid genotypes, enumerated as *AA*, *AC*, *AG*, *AT*, *A*–, *CC*, *CG*, *CT*, *C*–, *GG*, *GT*, *G*–, *TT*, *T*–, and −−. In general, the relation between *R*_*i*_ and *G*_*i*_, i.e., the state of base *i*, can be defined by “Match,” “SNP,” “Ins,” and “Del” and we use a latent variable *Z*_*i*_ to describe this hidden state on base *i*. Depending on the value that *R*_*i*_ takes, two cases should be considered for *Z*_*i*_:

1. If *R*_*i*_≠−, the state can be a “Match,” “SNP,” or “Del” and correspondingly the hidden state variable *Z*_*i*_ can take 15 possible values in accordance with the 15 diploid genotypes, denoted by *s*_*j*_,*j*=1,…,15. For example, suppose the reference base *R*_*i*_=*A*, then *s*_1_=*AA* representing the state “Match,” *s*_15_=−− representing the state “Del,” and other states may be considered as “SNP”s.
2. If *R*_*i*_=−, the state can be either an “Ins” or a not valid state and the hidden state variable *Z*_*i*_ can also take 15 values, denoted by *s*_*j*_,*j*=16,…,30. For example, suppose the reference base *R*_*i*_=−, then *s*_30_=−− is a not valid state, and all other states are considered as “Ins”.

It is worth noting that the inference of the most likely genotype *G*_*i*_ is equivalent to finding the most likely *Z*_*i*_, which directly indicates the occurrence of the variant—SNP or INDEL, on base *i*.

After defining the hidden states, we characterize transitions among the states by a transition matrix *T*={*t*_*mn*_},1≤*m*≤30,1≤*n*≤30. Each component of the matrix is defined by *t*_*mn*_=*P*(*Z*_*i*+1_=*n*|*Z*_*i*_=*m*),1≤*i*<*L*, representing the probability of being in state *n* at the current base given the observed state *m* at the previous base. In our simulation studies, these transition probabilities are set by empirical values. In the analysis of real data, the transition matrix can be obtained by calculating the conditional frequencies of the variants from the NCBI dbSNP database (version 136) [25].

### The emission probabilities

Emission probabilities govern the distribution of the observed data (a pileup of reads) at each base given the hidden state at that base. In vi-HMM, we first identify the bases on which the pileup of reads have size ≥5. Denoting these read data on base *i* by *D*_*i*_,1≤*i*≤*L*, we write the probability (likelihood) of observing *D*_*i*_ given the hidden state *Z*_*i*_ as

1 $$  P_{i}=L\left(Z_{i}|D_{i} \right) = \prod\limits_{k=1}^{n_{i}} p \left(d_{ik}|Z_{i} \right), \quad Z_{i} \in \left\{ s_{1},\ldots,s_{30}\right\}  $$

where *d*_*ik*_ represents the nucleotide on the *k*th read covering base *i* and *n*_*i*_ represents the size of the pileup on that base. Since each value taken by the hidden state *Z*_*i*_ corresponds to a specific underlying genotype *G*_*i*_ which contains two alleles *A*_1_ and *A*_2_, we further write the probability of observing each *d*_*ik*_,1≤*i*≤*L*,1≤*k*≤*n*_*i*_ given *Z*_*i*_ as

2 $$ \begin{aligned}  p\left(d_{ik}|Z_{i} \right) &= p \left(d_{ik}| \left\{ A_{1}, A_{2} \right\} \right) \\ &= \frac{1}{2}p \left(d_{ik}|A_{1} \right) + \frac{1}{2} p \left(d_{ik}|A_{2} \right) \end{aligned}  $$

where the probability of observing *d*_*ik*_ given one allele *A*∈{*A*_1_,*A*_2_} is

3 $$  p \left(d_{ik}|A \right) =\left\{ \begin{array}{ll} {e_{ik}} & \text{if}~ d_{ik}\neq A \\ 1-\frac{e_{ik}}{4} & \text{if}~ d_{ik}= A \end{array} \right..  $$

In the above expression (3), *e*_*ik*_ represents the sequencing error rate on base *i* for read *k*, which can be calculated from the reversed Phred-scaled quality score in the SAM file. For simplicity, here, we assume that the sequencing error on base *i* is caused by four possible point mutations (from allele *A* of *G*_*i*_ to the nucleotide of *d*_*ik*_ which may take four other symbols in set *Ω*) with equal probability. In particular, when “ −” appears in a read (meaning a deletion in the CIGAR string of the SAM file) so the corresponding Phred quality score on that read base is missing, we take the average of all other reads’ Phred quality scores on that base to impute the missing value.

We note that the emission distribution can vary for different bases. Given *n*_*i*_ pileup reads on base *i*, for each possible combination of *R*_*i*_ and *G*_*i*_, i.e., for *Z*_*i*_=*s*_*j*_,1≤*s*_*j*_≤30, the emission distribution at this base will be a discrete distribution which categorizes the pileup read data *D*_*i*_ into 15 groups corresponding to the possible diploid genotypes. Nevertheless, the probability of observing *D*_*i*_ given *Z*_*i*_ can be easily calculated through a multinomial probability mass function (PMF) by incorporating the sequencing error rates *e*_*ik*_,1≤*k*≤*n*_*i*_.

### The optimal state sequence

With the HMM parameters identified, we use the Viterbi algorithm to find the optimal hidden state path **Z**=*Z*_1_*Z*_2_…*Z*_*L*_, which not only indicates the most likely genotypes but also can be used to call SNPs and INDELs directly.

## Datasets

### Simulated data

To evaluate the performance of vi-HMM, we simulate two datasets of short reads using different processes: one introducing positional dependence by HMM and the other assuming random occurrence of the genetic variants by the wgsim tool [26]. In the first process, the simulation starts with generating a 50,000 base pairs (bp) genomic segment as the reference sequence. In order to take into account the spatial dependence in the genotype, we first generate a haplotype sequence based on an HMM with four states: “Match,” “SNP,” “Del,” and “Ins.” The transition matrix of this HMM is pre-specified (for details, please refer to https://github.com/tangmanhd/vi-HMM). The observed haplotype sequence (which takes value from the alphabetic set *Ω*) is determined by the emission distribution, which, for simplicity, is set to be discrete uniform conditioning on the hidden states. That is, for each base of the haplotype, the probability vector of observing a nucleotide symbol other than the corresponding nucleotide shown in the reference is [1/3,1/3,1/3] if the hidden state is “SNP” and is [1/4,1/4,1/4,1/4] if the hidden state is “Ins” (note that the emissions for the other two hidden states are deterministic). Based on the generated haplotype sequence, the second haplotype can be generated by incorporating a pre-specified heterozygous rate. Once the haplotype pair is generated, we then generate the short-read data, half from each haplotype, by specifying the length, number of reads, and base quality. In the second process, we randomly select a 50,000-bp segment of chromosome 21 on the human genome as the reference sequence and then use wgsim to simulate paired end reads (https://github.com/lh3/wgsim) (Additional file 2). In both processes, the base-calling errors are considered to be stochastic and are generated from a uniform distribution. These base-calling error probabilities are then transformed into Phred quality scores for sequence alignment and variant calling.

In each simulation process, four datasets were generated at the 15×, 20×, 25×, and 30× sequencing depths, respectively. The simulated short reads are, on average, 100 bp long. All simulated reads are mapped to the reference sequence by using sequence alignment tools Bowtie2 (version 2.2.5) and BWA-MEM (version 0.7.12). After read alignment, we apply vi-HMM, GATK HaplotypeCaller (version 4.0), FreeBayes (version 1.1.0), Platypus (version 0.8.1), SAMtools (version 1.3), and VarScan (version 2.3.9) to these datasets for variant calling (commands and settings for extant variant callers are listed in Additional file 2). Evaluation of the calling accuracy is based on the following criteria. For SNP calling, if the locus of a called SNP is exactly the same as the truth, this SNP is recorded as a true positive (TP); otherwise, it is a false positive (FP). On the other hand, if a true SNP is not identified by the caller, it is a false negative (FN). For INDEL calling, if the called locus is the same as the the simulated truth, this INDEL is regarded as a TP, and the definitions of FP and FN are the same as those for calling SNPs. With these concepts, we calculate the sensitivity, precision, and *F*_1_ score by:

4 $$ \begin{aligned} \text{sensitivity} &= \mathrm{\frac{TP}{TP + FN}}\\ \text{precision} &= \mathrm{\frac{TP}{TP + FP}}\\ F_{1} &= \mathrm{\frac{2 TP}{2 TP + FP + FN}} \end{aligned}  $$

This simulation procedure is repeated 1000 times to summarize the averages on sensitivity, precision, and *F*_1_ score.

### Real data

We further run vi-HMM to call SNPs and INDELs on a dataset from the GIAB project (NA12878; chr21, 1–48129895 and chr22, 1–51304566; genome version hs37d5). This dataset consists of 19,020,457 (chr21) and 17,598,950 (chr22) mapped reads. The average lengths of reads on chr21 and chr22 are 100.9 bp and 101 bp and the average coverages on chr21 and chr22 are 54× and 50×, respectively. In order to evaluate the performance of variant calling at lower coverage, we downsample this real dataset to 15× and 30× sequencing depths and then apply several variant callers to the datasets accordingly. These three datasets are denoted as low (15×), medium (30×), and high (54× or 50×) coverage depths, respectively. A validation dataset by Zook et al. [22] is treated as the “ground truth” to evaluate the calling accuracy [22]. It should be noted that Zook et al. [22] has applied GATK in the process of obtaining these high-confidence variants. Therefore, to avoid biased comparison, we choose to not include GATK but only compare the vi-HMM calling results to those generated by the other four popular variant callers—FreeBayes, Platypus, SAMtools, and VarScan (version numbers of these callers are the same as those in simulations). The transition matrix of vi-HMM is pre-specified according to the conditional frequencies estimated from the NCBI dbSNP database (version 136) [25].

## Results

### Performance evaluation based on data simulated by HMM

**Fig. 2:**
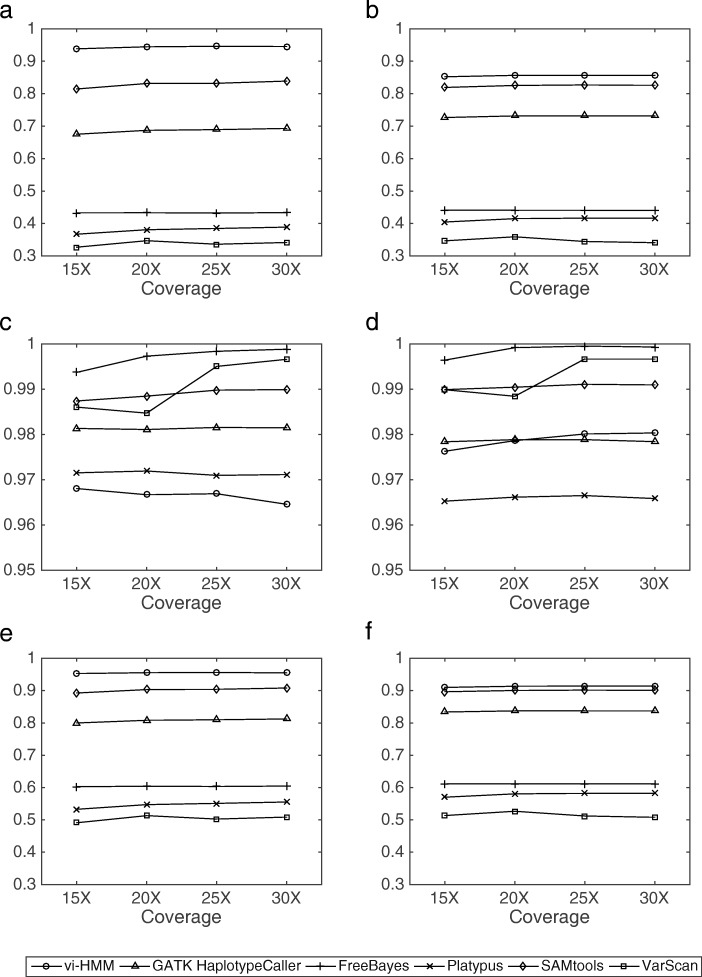
Comparison of SNP calling by different variant callers using data simulated by HMM at various sequencing depths. **a** Sensitivity with Bowtie2 mapping. **b** Sensitivity with BWA-MEM mapping. **c** Precision with Bowtie2 mapping. **d** Precision with BWA-MEM mapping. **e** *F*_1_ score with Bowtie2 mapping. **f** *F*_1_ score with BWA-MEM mapping

The SNP calling results by the six variant callers (vi-HMM, GATK HaplotypeCaller, FreeBayes, Platypus, SAMtools, and VarScan) for the simulated data are shown in Fig. 2. We observe that, when Bowtie2 is used for read mapping, vi-HMM achieves the highest sensitivity and *F*_1_ score at every read coverage depth, indicating its good accuracy in detecting SNPs as compared to the other variant callers, especially at the low-coverage (15× depth) setting (Fig. 2a, e). The sensitivity of SNP calling by vi-HMM reaches 93.83%, whereas the second highest sensitivity by SAMtools is only 81.45% at the 15× depth; the *F*_1_ score by vi-HMM (95.29%) is also much higher than that by SAMtools (89.27%). All six variant callers show high precision (above 95%) on this simulated data (Fig. 2c). When BWA-MEM is used for read mapping, the sensitivities and *F*_1_ scores by vi-HMM are also the highest across all read coverage depths (Fig. 2b, f). The sensitivity and *F*_1_ score by SAMtools are comparable to those by vi-HMM. Again, high precision is observed for all six variant callers (Fig. 2d).

**Fig. 3:**
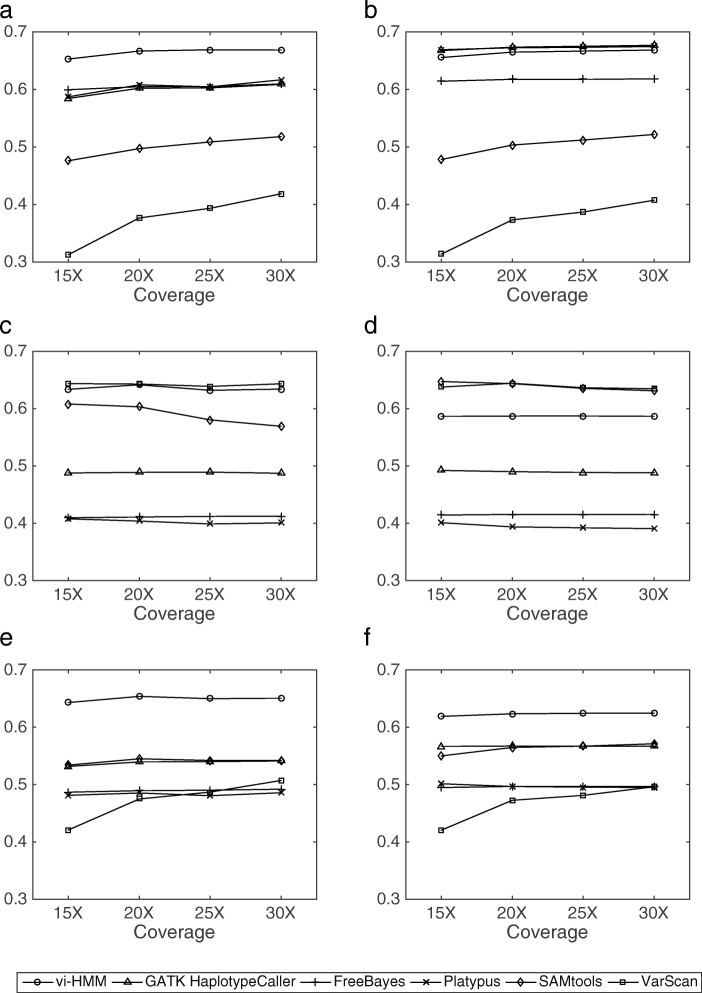
Comparison of INDEL calling by different variant callers using data simulated by HMM at various sequencing depths. **a** Sensitivity with Bowtie2 mapping. **b** Sensitivity with BWA-MEM mapping. **c** Precision with Bowtie2 mapping. **d** Precision with BWA-MEM mapping. **e** *F*_1_ score with Bowtie2 mapping. **f** *F*_1_ score with BWA-MEM mapping

For INDEL calling, with Bowtie2 mapping, the sensitivity and *F*_1_ score by vi-HMM are the highest at every read coverage depth (Fig. 3a, e) and the precision by vi-HMM is the second highest (Fig. 3c), indicating the superiority of vi-HMM in detecting INDELs than the other variant callers. As the coverage depth increases, the INDEL calling accuracy of vi-HMM becomes higher. With BWA-MEM mapping, the sensitivity by vi-HMM is only slightly lower than those by Platypus and GATK HaplotypeCaller (Fig. 3b). The precisions by vi-HMM are much higher than those by GATK HaplotypeCaller, FreeBayes, and Platypus and slightly lower than those by the other methods (Fig. 3d). Overall, the *F*_1_ score by vi-HMM reaches the highest at every read coverage depth (Fig. 3f).

### Performance evaluation based on data simulated by wgsim

**Fig. 4:**
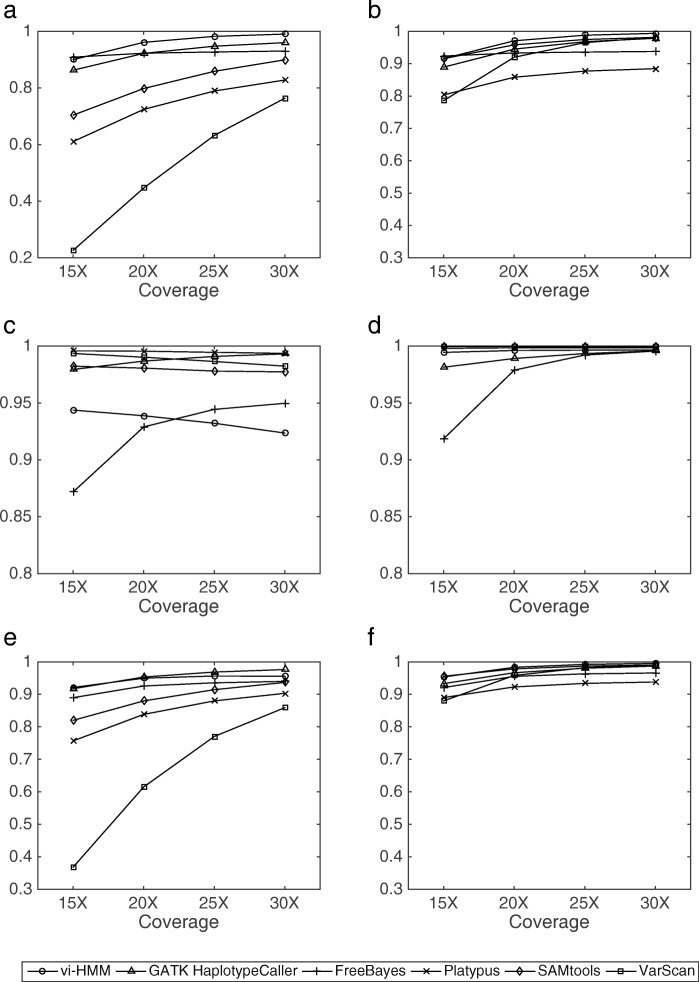
Comparison of SNP calling by different variant callers using data simulated by wgsim at various sequencing depths. **a** Sensitivity with Bowtie2 mapping. **b** Sensitivity with BWA-MEM mapping. **c** Precision with Bowtie2 mapping. **d** Precision with BWA-MEM mapping. **e** *F*_1_ score with Bowtie2 mapping. **f** *F*_1_ score with BWA-MEM mapping

**Fig. 5:**
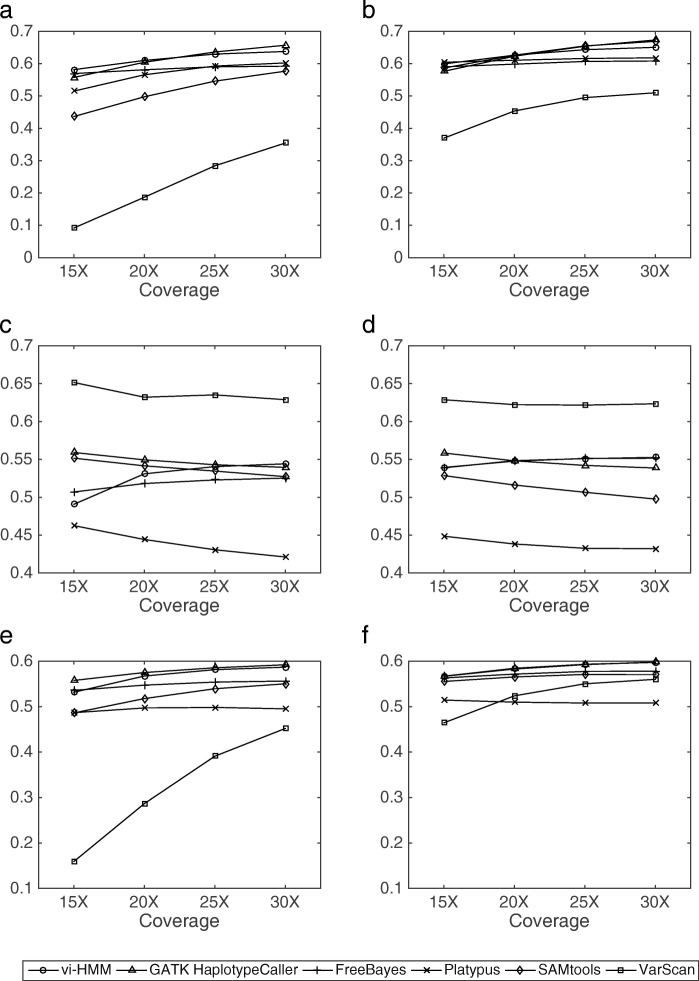
Comparison of INDEL calling by different variant callers using data simulated by wgsim at various sequencing depths. **a** Sensitivity with Bowtie2 mapping. **b** Sensitivity with BWA-MEM mapping. **c** Precision with Bowtie2 mapping. **d** Precision with BWA-MEM mapping. **e** *F*_1_ score with Bowtie2 mapping. **f** *F*_1_ score with BWA-MEM mapping

In general, vi-HMM performs well in calling SNPs and INDELs on the data simulated by wgsim. For SNP calling, when Bowtie2 is used for read mapping, the sensitivity by vi-HMM is slightly lower than that by FreeBayes at the low-coverage (15× depth) setting but becomes the highest when the read coverage depth increases (Fig. 4a). *F*_1_ scores by vi-HMM and GATK HaplotypeCaller are the highest across all read coverage depths, with only subtle differences between the two (Fig. 4e). When BWA-MEM is used for read mapping, the sensitivity by vi-HMM is the highest at the medium to high-coverage (20×, 25×, 30× depths) settings (Fig. 4b). The *F*_1_ scores by vi-HMM and SAMtools are the highest at every read coverage depth (Fig. 4f). For INDEL calling, the sensitivity by vi-HMM reaches the highest at 15× and 20× depths on reads mapped with Bowtie2 (Fig. 5a). Under both mapping methods, the *F*_1_ scores by vi-HMM and GATK HaplotypeCaller remain the highest when the read coverage depth increases (Fig. 5e, f).

### Application to the real data for NA12878

The results of comparing the sensitivity, precision, and *F*_1_ score between the five variant callers are shown in Table 1, by using real data on chr21 at the 15×, 30×, and 54× sequencing depths. For SNP calling, we observe that all five callers except FreeBayes achieve very high precision (>99*%*) at the three depths. Thus, the differences in *F*_1_ score are mainly driven by sensitivity, for which vi-HMM and SAMtools outperform the others especially at low (15×: both >95*%*) to medium (30×: both >99*%*) depths. For INDEL calling, it is obvious that vi-HMM produces the highest *F*_1_ score over all other callers, and the superiority in *F*_1_ score becomes more apparent at low (15×: vi-HMM >91*%* whereas others <90*%*) to medium depths (30×: vi-HMM >95*%* whereas other callers’ *F*_1_ scores range from 80.54 to 93.67*%*). We also note that among all five variant callers, vi-HMM is able to control the false positives and false negatives in a balanced way (i.e., achieve >90*%* sensitivity and precision simultaneously) for both SNP and INDEL calling at all three depths, whereas others cannot (for example, FreeBayes and Platypus have low precision in INDEL calling, the sensitivity of SAMtools for calling INDELs is less competitive, and the sensitivity of VarScan for calling both SNPs and INDELs drops too fast at lower depths). To check the consistency of the comparison results on different chromosomes, we also apply the same variant calling process to chromosome 22. Similarly, for SNP calling, vi-HMM and SAMtools achieve very high *F*_1_ score at low (15×: both >96*%*) to medium (30×: both >99*%*) depths, and for INDEL calling, vi-HMM also outperforms the others at low to medium depths (at low depth, the *F*_1_ scores of vi-HMM and SAMtools are comparable, see details in Additional file 3). These comparisons provide us evidence that on the real datasets, vi-HMM represents an improvement over the other four variant callers in terms of calling SNPs and INDELs, as its performance gets closer to the recognized “ground truth”—which was obtained by GATK in practice.

**Table 1:** Comparison of different variant callers using real data on chromosome 21

	SNP			INDEL		
Caller	Sensitivity (%)	Precision (%)	*F*_1_ score (%)	Sensitivity (%)	Precision (%)	*F*_1_ score (%)
15 ×						
vi-HMM	95.11	99.62	97.31	91.95	90.18	91.06
FreeBayes	94.82	91.61	93.18	88.93	74.79	81.25
Platypus	90.97	99.84	95.20	93.74	70.03	80.17
SAMtools	98.66	99.56	99.11	83.79	95.45	89.24
VarScan	76.31	99.87	86.51	74.00	99.44	84.85
30 ×						
vi-HMM	99.81	99.44	99.63	95.22	95.62	95.42
FreeBayes	95.80	95.48	95.64	90.36	76.41	82.80
Platypus	92.92	99.73	96.21	95.67	69.54	80.54
SAMtools	99.64	99.62	99.62	87.84	93.23	90.46
VarScan	97.93	99.82	98.86	88.59	99.37	93.67
54 ×						
vi-HMM	99.95	99.18	99.56	95.61	96.09	95.85
FreeBayes	95.88	96.90	96.39	90.77	77.27	83.48
Platypus	92.97	99.63	96.18	96.06	69.11	80.38
SAMtools	99.70	99.61	99.65	88.99	90.53	89.75
VarScan	99.53	99.77	99.65	91.67	99.24	95.31

## Discussion

In this article, we describe a new HMM-based method, vi-HMM, for accurate calling of SNP and INDEL variants in mapped reads. By taking advantage of the HMM features, vi-HMM allows us to detect variants directly through inferring an optimal hidden state path from the observed pileup read data and the reference genome. Both simulation studies and real data analysis have confirmed that vi-HMM is able to improve the accuracy of SNP/INDEL identification as compared to other variant callers, especially at low and medium depths.

As an important step in NGS data analysis, variant calling has received much attention in bioinformatics research. Although a number of variant calling methods have been developed, it remains unclear how different model assumptions used in these methods affect their practical performance. In general, the performance of a variant caller can be evaluated through either real data analysis or simulations. Real data analysis is able to reveal features of the variant caller under different settings (sequencing platforms, coverage depths, etc), however, due to lack of “ground truth” on experimentally validated variant sets in real data, the results of false positives and false negatives in variant identification are often arguable. Simulation studies, on the other hand, provide strong evidences for evaluation of a variant caller or comparison among variant callers. However, the simulated data need to be justified to have similar characteristics as real data in order to guarantee that the conclusions still remain meaningful in real data scenarios.

In the present work, we have performed both simulations and real data analysis to evaluate the proposed variant caller vi-HMM and compare it with other commonly used callers. Interestingly, we found something in common in the two sets of calling results (at 15× and 30× depths using both simulated and real data): (1) Overall, vi-HMM and SAMtools have higher *F*_1_ score than FreeBayes, Platypus, and VarScan, in both SNP calling and INDEL calling. (2) The precision for most variant callers are very high in SNP calling. (3) When sequencing depth increases from low (15×) to medium (30×), most variant callers have better calling performance. (4) The sensitivity and precision for vi-HMM are balanced and remain high across different depths, whereas for the other variant callers they could be very unbalanced (e.g., Platypus and FreeBayes in INDEL calling) or easily influenced by low depth of the data (e.g., the fast dropping of VarScan sensitivity in INDEL calling from 30× to 15×). These findings in variant calling performance indicate that our simulated data share some similarities with the real data, and both demonstrate that our proposed method, vi-HMM, has a good performance overall and is applicable not only to med-to-high read coverage depth but also to low read coverage, with robust performance.

Particularly, for the two “better performers” vi-HMM and SAMtools, we also see the differences between their SNP calling and INDEL calling. While they both have high sensitivity, precision, and *F*_1_ score in simulations and real data analysis, vi-HMM does not display remarkable superiority in calling SNPs. This may be because the state “SNP” is more likely to move to “Match” (94.99*%* from dbSNP) rather than to another variant across the genome. Thus the dependence between “SNP” and the adjacent variants becomes negligible and plays a less important role in SNP calling. However, in terms of INDEL calling, vi-HMM certainly outperforms SAMtools. This could be possibly explained by the fact that the transition probability from the state “Ins” to “Match” is only 28.80*%* (data from dbSNP), indicating that there exists strong dependence between “Ins” and the adjacent variants and therefore vi-HMM should have a better performance in calling INDELs by considering such state dependence between adjacent genomic bases.

**Fig. 6:**
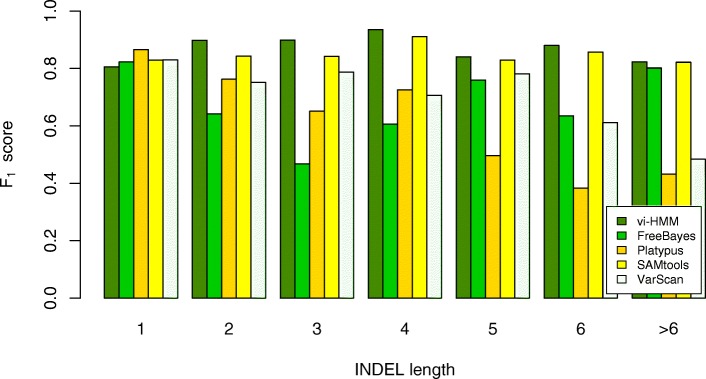
*F*_1_ scores by vi-HMM, FreeBayes, Platypus, SAMtools, and VarScan at different INDEL lengths on real data with 15× depth on chromosome 21

Another observation in real data analysis is that, the *F*_1_ scores of these tools vary at different INDEL lengths. Figure 6 shows the *F*_1_ scores by vi-HMM, FreeBayes, Platypus, SAMtools, and VarScan at INDEL lengths 1, 2,..., 6, and >6 on real data with 15× depth on chromosome 21. We see that the *F*_1_ score by vi-HMM remains above 80% for all INDEL lengths whereas other variant callers, such as FreeBayes, Platypus, and VarScan cannot maintain their *F*_1_ scores consistently high. In particular, all these tools have comparable *F*_1_ scores at INDEL length 1, and vi-HMM achieves the highest *F*_1_ score at INDEL length from 2 to 6, indicating that this HMM-based method appears to be more accurate in detecting short INDELs.

Noteworthy, the accuracy of variant calling also depends on the quality of read alignment. In general, the occurrence of INDELs in reads may shift the alignments and result in mismatch [27], which may impact the subsequent variant calling procedure remarkably. This is especially true for large INDELs. As seen from our simulation study 1, vi-HMM produces higher sensitivity and *F*_1_ score on reads mapped with Bowtie2 than it does on reads mapped with BWA-MEM at every read coverage depth. One plausible explanation is that Bowtie2 performs better than BWA-MEM in the read alignment (further examination of the two aligners on correct mapping, multiple alignment, second alignment, soft/hard clipped reads is included in Additional file 4). Such a phenomenon of variant calling being influenced by read alignment can also be observed in a simulated dataset with homopolymers (Additional file 5). It is thus important to choose an alignment tool that produces high-quality mapping prior to variant calling.

## Conclusion

In conclusion, we have developed a novel HMM-based method for sequence variant identification in short-read data. This variant caller provides an effective solution to modeling the dependence of adjacent genomic loci, which is expected to be useful for accurate calling of variants but is often overlooked in existing tools. To evaluate the performance of calling SNPs and INDELs in synthetic and real sequencing data, we compared the new variant calling method, vi-HMM, with five prevalent methods (GATK HaplotypeCaller, FreeBayes, Platypus, SAMtools, and VarScan) in simulation studies and with four (FreeBayes, Platypus, SAMtools, and VarScan) in real data analysis. Both comparison results demonstrate that vi-HMM is able to identify SNP and INDEL variants in a more accurate (overall high *F*_1_ score), reliable (smaller fluctuations across different read coverage depths), and balanced (both good sensitivity and good precision) way, as compared to the other variant callers.

## Additional files

Additional file 1 Mapped reads in IGV viewer. (PDF 78 kb)

Additional file 2 Commands and settings for data simulation by wgsim and variant calling by GATK HaplotypeCaller, FreeBayes, Platypus, SAMtools, and VarScan. (PDF 77 kb)

Additional file 3 Performance of different variant callers using real data on chromosome 22. (PDF 83 kb)

Additional file 4 The alignment information by Bowtie2 and BWA-MEM at different coverage depths. (PDF 36 kb)

Additional file 5 Performance of vi-HMM on simulated data with homopolymers. (PDF 60 kb)
