# Supplementary material for: vi-HMM: a novel HMM-based method for sequence variant identification in short-read data
Source: Hum Genomics. 2019 Feb 13;13:9. doi: 10.1186/s40246-019-0194-6 (PMC6387560; doi:10.1186/s40246-019-0194-6)
Supplement: Supplementary file 1 — Mapped reads in IGV viewer. (PDF 78 kb) [file 40246_2019_194_MOESM1_ESM.pdf]

Additional file 1:

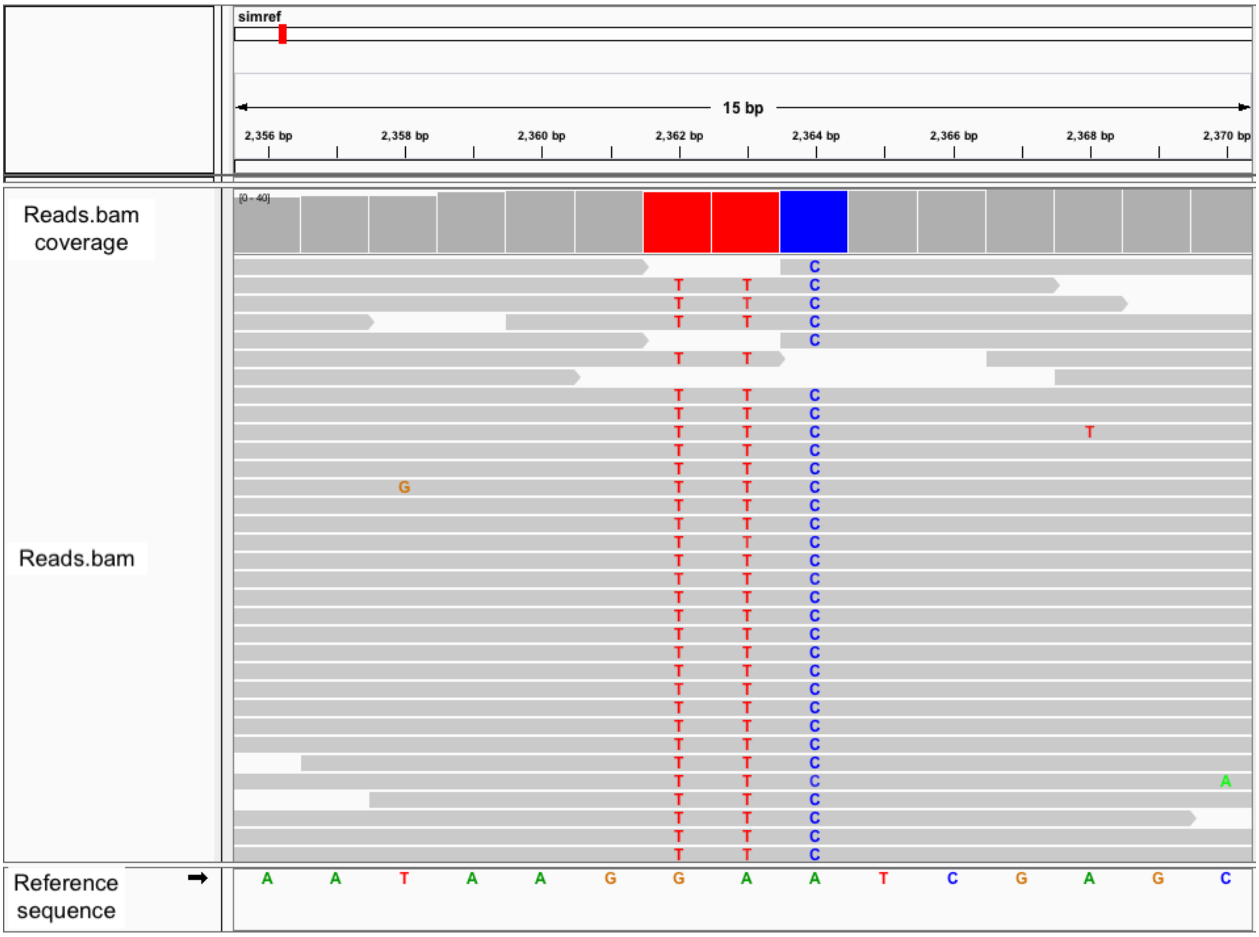

Figure 1: SNPs in IGV viewer. This figure illustrates a view of a section of mapped reads in the dataset simulated by HMM. It shows three SNPs at base 2362 (G $\rightarrow$ T), 2363 (A $\rightarrow$ T) and 2364G (A $\rightarrow$ C).
