# Supplementary material for: vi-HMM: a novel HMM-based method for sequence variant identification in short-read data
Source: Hum Genomics. 2019 Feb 13;13:9. doi: 10.1186/s40246-019-0194-6 (PMC6387560; doi:10.1186/s40246-019-0194-6)
Supplement: Supplementary file 2 — Commands and settings for data simulation by wgsim and variant calling by GATK HaplotypeCaller, FreeBayes, Platypus, SAMtools, and VarScan. (PDF 77 kb) [file 40246_2019_194_MOESM2_ESM.pdf]

Additional file 2:

Data simulation and variant calling are performed by using the following commands.

#1. Simulate short read data by wgsim

The number of read pairs should be set according to the read coverage depth. For repeated simulations, the Seed should be given different values so that different sets of variants can be generated in each simulated data.

```
wgsim -e 0.0175 -d 500 -s 50 -N <number of read pairs> -1 100 -2
100 -r 0.01 -R 0.15 -X 0.30 -S <Seed> <reference.fasta> <out.read1.fq>
<out.read2.fq> 1> <variants.txt>
```

#2. Call variants by GATK HaplotypeCaller

```
java -jar GenomeAnalysisTK.jar -T HaplotypeCaller -R <reference.fasta>
-I <input.bam> -o <output.vcf>
```

#3. Call variants by FreeBayes

```
freebayes -f <reference.fasta> <input.bam> > <output.vcf>
```

#4. Call variants by Platypus

```
python Platypus.py callVariants --bamFiles=<input.bam> --refFile=<input.bam
> --output=<output.vcf>
```

#5. Call variants by SAMtools

```
samtools mpileup -g -f <reference.fasta> <input.bam> > <output.bcf>
bcftools call -c -v <output.bcf> > <output.vcf>
```

#6. Call variants by VarScan

```
samtools mpileup -f <reference.fasta> <input.bam> > <output.mpileup>
```

##SNP

```
java -jar VarScan.v2.3.9.jar mpileup2snp <output.mpileup> --output-vcf
1 > <output.vcf>
```

##INDEL

```
java -jar VarScan.v2.3.9.jar mpileup2indel <output.mpileup> --output-vcf
1 > <output.vcf>
```
