# Supplementary material for: vi-HMM: a novel HMM-based method for sequence variant identification in short-read data
Source: Hum Genomics. 2019 Feb 13;13:9. doi: 10.1186/s40246-019-0194-6 (PMC6387560; doi:10.1186/s40246-019-0194-6)
Supplement: Supplementary file 3 — Performance of different variant callers using real data on chromosome 22. (PDF 83 kb) [file 40246_2019_194_MOESM3_ESM.pdf]

Additional file 3:

Comparison of different variant callers using real data on chromosome 22.

	SNP			INDEL		
Caller	Sensitivity	Precision	F_1 score	Sensitivity	Precision	F_1 score
**15X**						
vi-HMM	94.19%	99.36%	96.71%	89.79%	77.72%	83.32%
FreeBayes	94.75%	82.48%	88.19%	87.83%	63.83%	73.93%
Platypus	90.87%	99.86%	95.15%	92.84%	62.41%	74.64%
SAMtools	98.53%	99.58%	99.05%	77.99%	90.62%	83.83%
VarScan	72.47%	99.94%	84.02%	64.60%	98.50%	78.03%
**30X**						
vi-HMM	99.80%	99.24%	99.52%	94.01%	89.89%	91.90%
FreeBayes	95.86%	90.15%	92.92%	89.66%	67.88%	77.27%
Platypus	93.26%	99.59%	96.32%	95.78%	61.21%	74.69%
SAMtools	99.74%	99.64%	99.69%	82.15%	88.24%	85.09%
VarScan	97.69%	99.87%	98.77%	84.29%	98.42%	90.81%
**50X**						
vi-HMM	99.96%	98.55%	99.25%	93.88%	89.98%	91.90%
FreeBayes	95.94%	92.98%	94.44%	89.54%	70.12%	78.65%
Platypus	93.37%	99.44%	96.31%	96.33%	60.49%	74.32%
SAMtools	99.80%	99.66%	99.73%	83.67%	85.34%	84.50%
VarScan	99.58%	99.84%	99.71%	88.93%	99.17%	93.32%
