# Supplementary material for: vi-HMM: a novel HMM-based method for sequence variant identification in short-read data
Source: Hum Genomics. 2019 Feb 13;13:9. doi: 10.1186/s40246-019-0194-6 (PMC6387560; doi:10.1186/s40246-019-0194-6)
Supplement: Supplementary file 4 — The alignment information by Bowtie2 and BWA-MEM at different coverage depths. (PDF 36 kb) [file 40246_2019_194_MOESM4_ESM.pdf]

Additional file 4:

Bowtie2 and BWA-MEM are compared on correct mapping, multiple alignment, second alignment, and soft/hard clipped reads. We do not list the average percentages of hard clipped reads because all of them are zeros for both aligners.

Table 5.1: The average percentage of correct mapping by Bowtie2 and BWA-MEM using simulated data at various sequencing depths.

Mapper	15X	20X	25X	30X
Bowtie2	96.31%	95.79%	96.35%	95.90%
BWA-MEM	94.69%	94.62%	94.65%	94.61%

Table 5.2: The average percentage of multiple alignment by Bowtie2 and BWA-MEM using simulated data at various sequencing depths.

Mapper	15X	20X	25X	30X
Bowtie2	3.15%	3.21%	3.15%	3.23%
BWA-MEM	0	0	0	0

Table 5.3: The average percentage of soft clipped reads by Bowtie2 and BWA-MEM using simulated data at various sequencing depths.

Mapper	15X	20X	25X	30X
Bowtie2	0	0	0	0
BWA-MEM	8.68%	8.74%	8.71%	8.76%
