# Supplementary material for: vi-HMM: a novel HMM-based method for sequence variant identification in short-read data
Source: Hum Genomics. 2019 Feb 13;13:9. doi: 10.1186/s40246-019-0194-6 (PMC6387560; doi:10.1186/s40246-019-0194-6)
Supplement: Supplementary file 5 — Performance of vi-HMM on simulated data with homopolymers. (PDF 60 kb) [file 40246_2019_194_MOESM5_ESM.pdf]

# Additional file 5:

We test the performance of vi-HMM with homopolymers on a simulated dataset with 30× coverage depth. vi-HMM produces high calling accuracies of SNPs and INDELs, which are only slightly smaller than those in the general situation. One potential explanation for the lower INDEL calling accuracy is that the INDELs are redundant in the output[1].

SNP and INDEL callings by vi-HMM using simulated data with homopolymers at 30× depths.

| | SNP | | | INDEL | | |
|---|---|---|---|---|---|---|
| Caller | Sensitivity | Precision | $F_1$ score | Sensitivity | Precision | $F_1$ score |
| Bowtie2 | 88.39% | 87.46% | 87.92% | 59.16% | 57.47% | 58.30% |
| BWA-MEM | 79.76% | 88.52% | 83.91% | 58.13% | 53.14% | 55.52% |

# References


[1] Hasan, M.S., Wu, X., Watson, L.T., Li, Z., Zhang, L.: Ups-indel: A better approach for finding indel redundancy. In: Computational Advances in Bio and Medical Sciences (ICCABS), 2016 IEEE 6th International Conference On, pp. 1–1 (2016). IEEE
